# Lactate Promotes Myoblast Differentiation and Myotube Hypertrophy via a Pathway Involving MyoD In Vitro and Enhances Muscle Regeneration In Vivo

**DOI:** 10.3390/ijms19113649

**Published:** 2018-11-19

**Authors:** Sakuka Tsukamoto, Ayako Shibasaki, Ayano Naka, Hazuki Saito, Kaoruko Iida

**Affiliations:** 1 Department of Nutrition and Food Science, Graduate School of Humanities and Sciences, Ochanomizu University, 2-1-1 Otsuka, Bunkyo, Tokyo 112-8610, Japan; g1570506@edu.cc.ocha.ac.jp (S.T.); ayk.shibasaki@gmail.com (A.S.); g1740529@edu.cc.ocha.ac.jp (H.S.); 2 Laboratory of Applied Nutrition, Faculty of Human Life and Environmental Sciences, Ochanomizu University, Tokyo 112-8610, Japan.; naka.a901@gmail.com; 3 The Institute for Human Life Innovation, Ochanomizu University, 2-1-1 Otsuka, Bunkyo-ku, Tokyo 112-8610, Japan

**Keywords:** lactate, muscle differentiation, fiber hypertrophy, MyoD, myosin heavy chain

## Abstract

Lactate is a metabolic substrate mainly produced in muscles, especially during exercise. Recently, it was reported that lactate affects myoblast differentiation; however, the obtained results are inconsistent and the in vivo effect of lactate remains unclear. Our study thus aimed to evaluate the effects of lactate on myogenic differentiation and its underlying mechanism. The differentiation of C2C12 murine myogenic cells was accelerated in the presence of lactate and, consequently, myotube hypertrophy was achieved. Gene expression analysis of myogenic regulatory factors showed significantly increased myogenic determination protein (MyoD) gene expression in lactate-treated cells compared with that in untreated ones. Moreover, lactate enhanced gene and protein expression of myosin heavy chain (MHC). In particular, lactate increased gene expression of specific MHC isotypes, MHCIIb and IId/x, in a dose-dependent manner. Using a reporter assay, we showed that lactate increased promoter activity of the MHCIIb gene and that a MyoD binding site in the promoter region was necessary for the lactate-induced increase in activity. Finally, peritoneal injection of lactate in mice resulted in enhanced regeneration and fiber hypertrophy in glycerol-induced regenerating muscles. In conclusion, physiologically high lactate concentrations modulated muscle differentiation by regulating MyoD-associated networks, thereby enhancing MHC expression and myotube hypertrophy in vitro and, potentially, in vivo.

## 1. Introduction

Decreases in muscle volume contribute to a number of adverse health outcomes, such as disability and frailty in the elderly [1,2] and various chronic diseases, including diabetes and obesity [3,4]. This is because muscle tissues are the major sites of glucose uptake and energy utilization. Therefore, it is important to increase muscle mass, not only for athletes but also for many other people such as the elderly, who often have chronic diseases. In clinical settings, anaerobic exercise, such as resistance training, is one of the most effective strategies to induce muscle hypertrophy. It is widely accepted that the main mechanism for inducing muscle hypertrophy by exercise training is increased muscle protein synthesis in muscle fibers, and much evidence also supports the involvement of muscle satellite cells in the process of training-induced muscle hypertrophy [5].

Muscle satellite cells, located between the basal lamina and sarcolemma of the muscle, are precursor cells with the potential to differentiate into mature muscle cells. These myogenic progenitor cells are normally in a quiescent state, although once stimulated, they become “activated” for the generation of new muscle fibers. Muscle damage is known to be a potent stimulator of satellite cells, inducing them to convert from a quiescent to an activated state. Activated satellite cells proliferate and then differentiate into mature muscle cells, ultimately regenerating newly formed myofibers to achieve muscle remodeling. This regenerative process is orchestrated by the expression of myogenic regulatory factors (MRFs), a group of bHLH transcription factors including myogenic factor 5 (Myf5), myogenic determination protein (MyoD), and myogenin [6,7]. In brief, Myf5 is initially expressed after satellite cells are activated and then MyoD and myogenin are sequentially expressed in the newly formed fibers. Terminal differentiation is achieved with the expression of structural and enzymatic muscle-specific proteins such as myosin heavy chain (MHC), the main motor protein in muscle filaments [8].

Although the exact role of satellite cells in muscle hypertrophy has been debated, there is direct evidence that satellite cells play a critical role in muscle remodeling and hypertrophy induced by resistance training and/or high-intensity exercise [9,10,11,12,13,14]. This leads to the question of what stimulates satellite cells to mediate muscle adaptation during and after exercise. Micro-injury arising from eccentric exercise should be a potent modulator of satellite cell activation [15]. However, lower-intensity training that might not cause muscle damage was also shown to lead to increases in the number of satellite cells [16]. Intriguingly, one study reported that short-term, low-load resistance exercise with partial blood flow restriction led to the marked proliferation of myogenic stem cells and the resulting addition of myonuclei in skeletal muscle [17]. Another study showed that hypoxia promoted satellite cell self-renewal and enhanced the efficiency of myoblast transplantation [18]. From these observations, we speculated that some muscle-derived mediators in anaerobic conditions, with lactate as a potential candidate, play a role. 

Lactate is a metabolic intermediate mainly produced in muscles under anaerobic conditions, especially during exercise [19,20]. It was previously regarded as “a metabolic waste product”, but is now known to be an important fuel source, either used within cells or exported to adjacent organs [19,20]. In addition, a recent publication suggested that lactate has signaling properties that regulate exercise-induced adaptations [21]. However, to date, few studies have examined the effects of lactate on myogenic cell biology. Willkomm et al. [22,23] showed that the expression of myogenin and MHC expression was decreased in lactate-treated C2C12 cells, a mouse muscle precursor cell line. In contrast, Oishi et al. [24] showed that the treatment of C2C12 cells with lactate resulted in increases in myogenin and the activation of p70S6K, a critical regulator of muscle protein synthesis. Both investigators also conducted in vivo studies, using human or mouse muscles harvested after exercise training, to confirm their results. However, in these experimental settings, the effects of lactate could not be comprehensively assessed. 

These inconsistent results and the lack of an in vivo study examining the direct effects of lactate on muscle remodeling prompted us to also investigate whether lactate influences myogenic differentiation. We analyzed the effects of lactate, with a focus on MRFs and their target proteins, in C2C12 cells. Furthermore, we assessed the physiological effects of elevated blood lactate on muscle remodeling and/or hypertrophy in a murine model involving peritoneal lactate injection. Our findings suggested that lactate plays a pathophysiological role in myogenic precursor cells via MyoD, a member of the MRFs, which contrasts with the results of previous studies.

## 2. Results

### 2.1. Lactate Promoted Myogenic Differentiation of C2C12 Myoblasts

**Figure 1:**
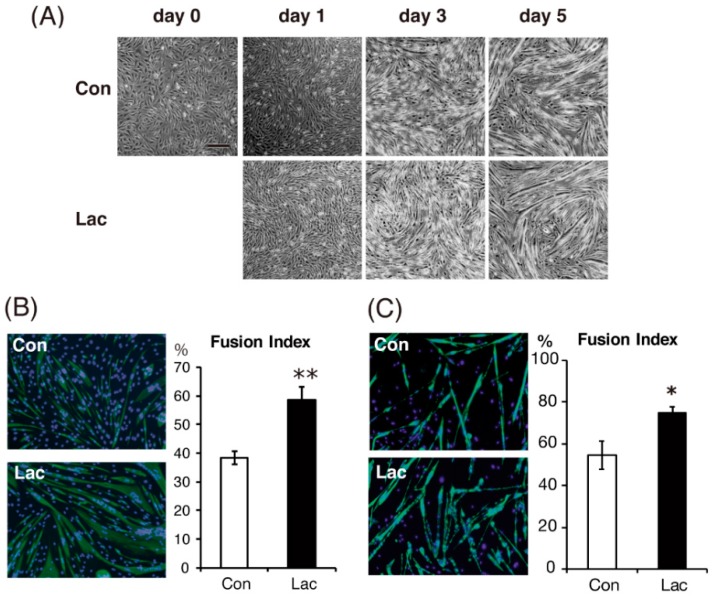
Lactate promoted myogenic differentiation of myoblasts. Cells were differentiated in control medium (Con) or medium containing 10 mM sodium lactate (Lac) for 5 d. (**A**) Microscopic images of C2C12 myoblasts after the induction of differentiation for the indicated days. Scale bar, 200 μm. (**B**,**C**) Differentiated C2C12 cells at day 5 (**B**) and primary myoblasts at day 3 (**C**) were stained with an MF-20 anti-myosin heavy chain (MHC) antibody (green) and 4′,6-diamidino-2-phenylindole (DAPI) (blue). Fusion index (%), defined as the percentage of nuclei located within MHC-positive myotubes divided by total nuclei, was calculated as described in Methods. *n* = 3–4 per group. Results are expressed as mean ± SE. * *p* < 0.05, ** *p* < 0.01 vs. Con.

We initially examined the effect of lactate on the morphological differentiation of C2C12 myoblasts. Considering the physiological range of blood levels, the concentration of lactate was initially set at 10 mM, which is seen in human blood and muscles after relatively high-intensity (65–85% W_max_) exercise [25]. When the cells were differentiated for 5 d with 10 mM lactate, we observed that myoblast fusion and myotube hypertrophy were accelerated, compared with untreated control cells (Figure 1A). The calculated fusion index values in C2C12 myotubes treated with 10 mM lactate were significantly higher (58.5% ± 4.4%) than in untreated myotubes (38.3 ± 2.4%) (Figure 1B). Lactate at 10 mM also enhanced myoblast fusion in primary cells assessed by fusion index at day 3 after the initiation of differentiation (Figure 1C).

### 2.2. MyoD and MHC Gene Transcription was Enhanced by Lactate

**Figure 2:**
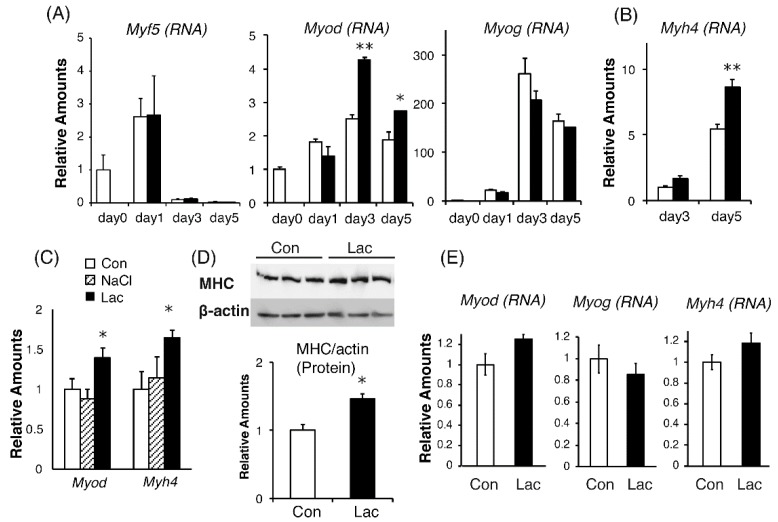
Lactate enhanced expression of *myogenic determination protein (Myod)* and *Myh4* genes and MHC protein in C2C12 myotubes. C2C12 cells (**A** to **D**) and primary myoblasts (**E**) were differentiated in control medium (open bar), or medium containing 10 mM sodium lactate (closed bar) or 10 mM sodium chloride (hatched bar). Cells were harvested on the indicated day of differentiation and messenger RNA (mRNA) expression levels of representative myogenic markers were determined by real-time PCR. (**A**) *Myf5*, *Myod*, and *Myog*; (**B**) *Myh4*; and (**C**) *Myod* and *Myh4*. RNA levels were quantified and normalized to that for GAPDH. Values are each expressed as the fold change compared with day 0 in (**A**), with control on day 3 in (**B**), and with control on day 5 in (**C**), with each the value used for normalization arbitrarily set to 1. (**D**) Cells were harvested on day 5. MHC protein levels were determined and normalized to those of β-actin. (**E**) Primary myoblasts were differentiated for 3 d and mRNA expression levels of *Myod*, *Myog*, and *Myh4* were determined. *n* = 3–4 per group. Results are expressed as means ± SE. * *p* < 0.05; ** *p* < 0.01; versus each control group.

As myogenic differentiation is strictly controlled by the sequential expression of MRFs, the levels of individual MRF genes were determined in time-dependent expression profiles [7,26], examining *Myf5*, *Myod*, and *Myog* (encoding myogenin) on days 1, 3, and 5. The expression levels of *Myf5* and *Myog* were not changed by 10 mM lactate, whereas *Myod* expression on days 3 and 5 was significantly increased (Figure 2A). *Myh4*, encoding myosin heavy chain (MHC) IIb, a highly abundant contractile protein in muscle, is generally used as a marker for the maturation of C2C12 myofibers [27]. During the differentiation of C2C12 cells, the levels of *Myh4* were elevated on days 3 and 5, and 10 mM lactate significantly enhanced its expression on both days (Figure 2B). Sodium chloride, at 10 mM, did not affect the expression of these genes (Figure 2C). This indicated that the lactate-induced changes in gene expression were not caused by sodium ions from the lactate sodium salt. Consistent with the results of measurements of gene expression, the augmentation of MHC protein expression by lactate was confirmed by Western blotting. C2C12 myotubes differentiated with 10 mM lactate contained more MHC protein than those differentiated without lactate (Figure 2D).

We also determined whether the lactate stimulation affected the expression of MRFs in the differentiation of primary myoblasts. Although statistically significant differences were not identified, similar trends were observed in the expression levels of *Myod*, *Myog*, *and Myh4* on day 3 of differentiation in these cells (Figure 2E).

**Figure 3:**
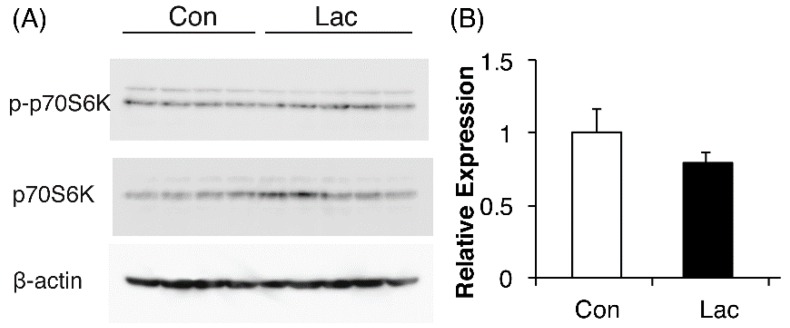
Lactate did not affect the protein synthesis pathway in C2C12 myotubes. (**A**) C2C12 cells were differentiated in control medium (Con) or medium containing 10 mM sodium lactate (Lac) for 5 d. Cells were harvested on day 5 and protein levels of P70S6K, p-P70S6K, and β-actin were analyzed by immunoblotting. (**B**) P70S6K phosphorylation levels (p-P70S6K/P70S6K) are expressed as fold change compared with that in the Con group (*n* = 4–5 per group). Results are expressed as mean ± SE.

A previous report showed that lactate increased the phosphorylation of ribosomal S6 kinase (P70S6K), a critical regulator of muscle protein synthesis [24]. We also examined whether prolonged treatment with lactate stimulated translation initiation and thereby increased MHC protein synthesis through p70S6K activation. We found that lactate had no effect on p70S6K activation, as assessed by phosphorylation (Figure 3).

### 2.3. Lactate Increased the Transcription of Specific MHC Isoforms in a Dose-Dependent Manner

**Figure 4:**
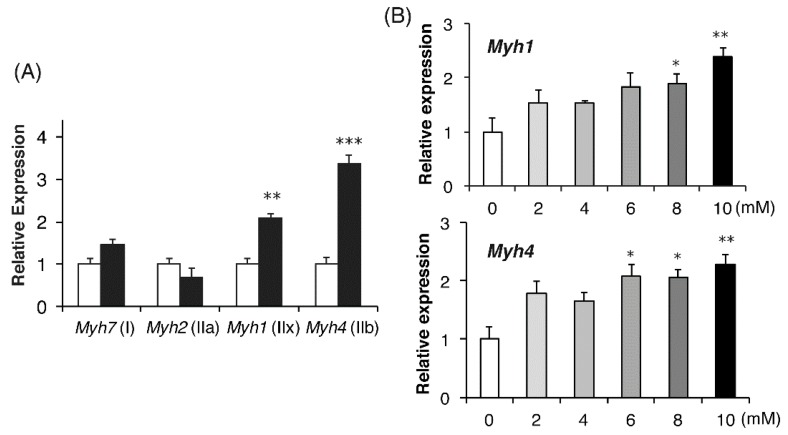
Lactate increased mRNA expression of predominantly the fast MHC isoform in C2C12 myotubes. (**A**) Cells were differentiated in control medium (open bar) or medium containing 10 mM sodium lactate (closed bar) for 5 d. Gene expression levels of *Myh7*, *Myh2*, *Myh1*, and *Myh4*, encoding MHC subtypes I, IIa, IIx, and IIb, respectively, were determined. Values are expressed as the fold changes, compared with each control (*n* = 4 per group). ** *p* < 0.01 versus the control. (**B**) Differentiation was induced in medium containing the indicated concentration of lactate. Cells were harvested on day 5 and expression levels of *Myh1* and *Myh4* were determined. Values are expressed as fold changes compared with the level in the 0 mM group (*n* = 4 per group). Results are expressed as mean ± SE. * *p* < 0.05, ** *p* < 0.01, *** *p* < 0.001, versus the 0 mM group.

As mammalian adult muscles contain four MHC isoforms (MHC I, IIa, IId/x, and IIb; although in human muscles, MHC IIb is barely detectable) [28], we next examined the effects of lactate on the expression of the genes encoding each isoform. Lactate stimulation for 5 d resulted in significantly increased expression of *Myh4* (encoding MHC-IIb) and *Myh1* (encoding MHC-IId/x) (Figure 4A). In contrast, the expression levels of *Myh2* (encoding MHC-IIa) and *Myh7* (encoding MHC-I) were not changed by lactate (Figure 4A). These effects of lactate on *Myh1* and *Myh4* expression were dose-dependent. Lactate levels above 8 and 6 mM significantly affected the expression of *Myh1* and *Myh4*, respectively, in C2C12 myotubes (Figure 4B).

### 2.4. Lactate-Induced Increases in Myh4 Promoter Activity Required Specific E-box Sequences

**Figure 5:**
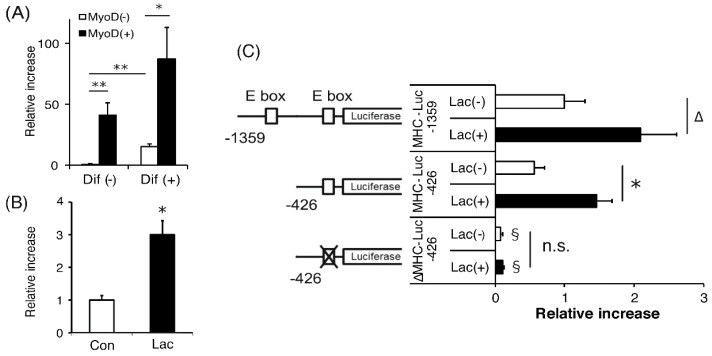
Lactate-induced increases in Myh4 promoter activity required specific E-box sequences. (**A**) Undifferentiated (Dif−) or differentiated (Dif+) C2C12 cells were cotransfected with a reporter vector containing 1.4 Kb of the Myh4 promoter region and a MyoD expression vector (pcDNA–MyoD; black bar) or its control (pcDNA3; white bar). (**B**) Differentiated C2C12 cells were transfected with a reporter vector containing the 1.4 Kb promoter region. At 24 h after transfection, the cells were incubated in the presence (Lac) or absence (Con) of 10 mM lactate for an additional 48 h. (**C**) Cells were then transfected with each deletion construct and then incubated in the presence (Lac+; black bar) or absence (Lac−; white bar) of 10 mM lactate for an additional 48 h. Luciferase activity was measured and is expressed as the fold induction corrected for transfection efficiency, based on *Renilla* luciferase activity. Values are expressed as the fold change compared with the vehicle control, which was arbitrarily set to 1. *n* = 4 per group. Results are expressed as mean ± SE. Δ *p* < 0.1, * *p* < 0.05, ** *p* < 0.01, § *p* < 0.05, versus the reporter vector containing 1.4 Kb of the promoter region

To investigate the effects of lactate on the transcriptional activity of MHC genes, reporter gene assays were performed, focusing on the MHC-IIb gene, *Myh4*. This is because, among the MHC isoforms, its expression was shown to be the most sensitive to lactate (Figure 4). The promoter activity of the region 1.4 Kb upstream of *Myh4* was significantly increased in C2C12 cells when differentiation was initiated, compared with that in the undifferentiated state. The reporter activity was further enhanced by forced MyoD expression in both undifferentiated and differentiated cells (Figure 5A), indicating that *Myh4* promoter activity was under the control of MyoD in C2C12 cells.

When C2C12 cells were differentiated in the presence of 10 mM lactate, a further increase in promoter activity was observed compared with that in its absence (Figure 5B). Therefore, using a series of promoter deletion constructs, we investigated the involvement of MRFs in the mechanism of the lactate-mediated increase in *Myh4* expression. A schematic diagram of each construct is shown in Figure 5C. Only 300 bp of the mouse *Myh4* proximal promoter is required to drive a reporter gene [29,30] and this region includes an E-box, which serves as the binding region for members of the MRF family [31], including MyoD. In addition, the 1.4 Kb promoter region used in our study contained another consensus E-box motif, located inside the myocyte-specific enhancer-binding nuclear factor 1 (MEF1) binding site [32]. The results showed that the activity of a deletion construct of the 0.4 Kb proximal promoter (without the MEF1 site) was still upregulated by lactate treatment for 48 h. However, deletion of only 6 bp of the proximal E-box led to significantly decreased promoter activity and also completely abolished the effects of lactate (Figure 5C).

### 2.5. Lactate Administration Accelerated Regeneration of Damaged Skeletal Muscle

**Figure 6:**
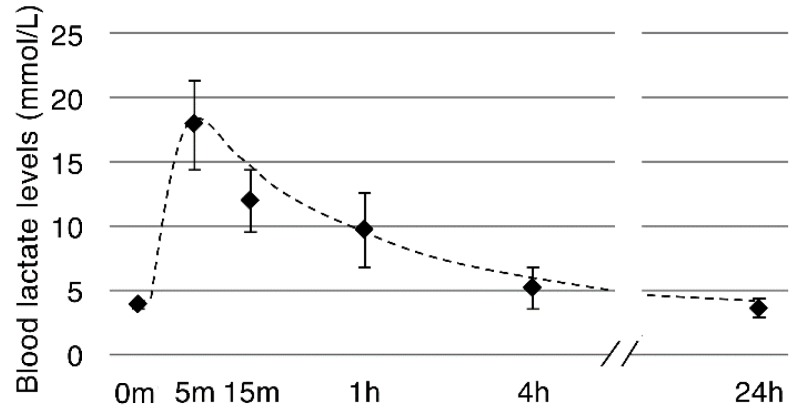
Blood lactate levels after intraperitoneal injection of lactate. Mice (eight weeks of age, *n* = 3) were intraperitoneally injected with sodium lactate (500 mg/kg). Blood lactate levels were measured with a blood lactate analyzer (Lactate Pro2; ARKRAY, Kyoto, Japan) at 0 min, 5 min, 15 min, 1 h, 4 h, and 24 h after injection. Results are expressed as mean ± SE.

An in vivo study was conducted to assess the effects of lactate on muscle regeneration and hypertrophy. Prior to the experiment, we confirmed the effects of the model on blood lactate using a few mice (*n* = 3), finding that the blood lactate levels were immediately increased from basal levels (3 to 4 mM) to about 15 mM within 5 min after intraperitoneal lactate injection (500 mg/kg) and were maintained at approximately 10 mM for at least 1 h (Figure 6).

**Figure 7:**
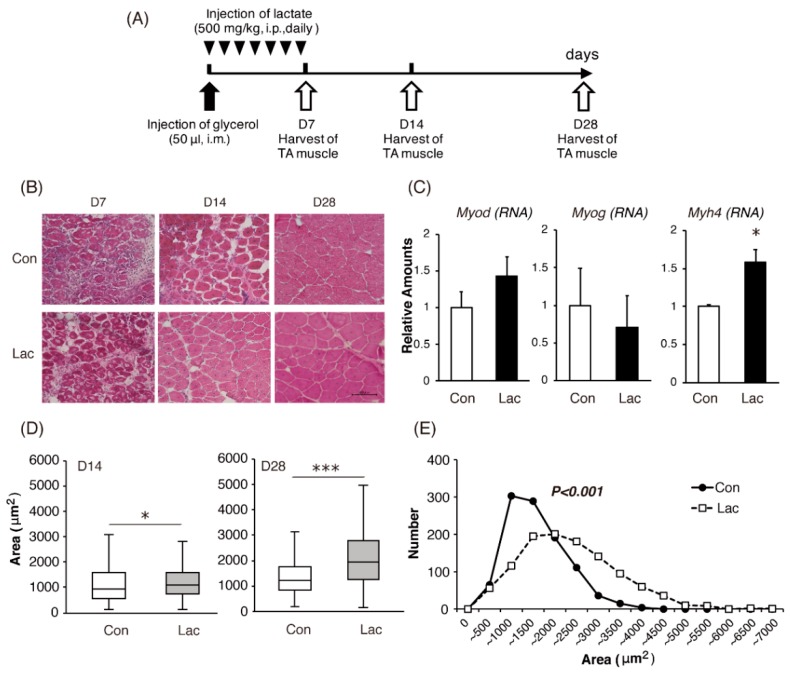
Lactate accelerated the regeneration of injured muscle. (**A**) Experimental scheme for the effects of lactate on damaged muscles induced by glycerol. (**B**) Representative images of hematoxylin and eosin-stained sections of tibialis anterior (TA) muscles from mice treated with lactate (Lac) or saline control (Con) at 7, 14, and 28 d after glycerol injection (scale bar = 100 µm). (**C**) Messenger RNA levels of representative myogenic markers were determined by real-time PCR in the harvested muscles at day 7. Results are expressed as mean ± SE. (**D**) Individual regenerated fiber areas were measured at day 14 and 28 in muscles from control (Con) and lactate-treated (Lac) mice (*n* = 3 per group). Values are presented in a box-and-whisker plot (boxes are constructed with the intervals between the first and third quartiles of the data distribution; lines in the boxes are medians; positive and negative bars are maximum and minimum individual values in each group, respectively. * *p* < 0.05, *** *p* < 0.001. (**E**) The distributions of fiber areas measured at day 28 muscles from control (Con; closed circle) and lactate-treated (Lac; open square) mice are shown.

The experimental design is summarized in Figure 7A. At day 7 after glycerol-induced injury, no obvious morphological differences were observed in the tibialis anterior (TA) muscle between the groups (Figure 7B; D7). However, the gene expression analysis revealed significant increases in the expression of *Myh4* and trends toward increased expression of *Myod*, but not of *Myog*, in TA muscles of mice in the lactate group (Lac), compared with that in the control (Con) (Figure 7C). Necrotic muscle fibers in both groups were replaced with abundant regenerating fibers by day 14 after glycerol injection (Figure 7B; D14), and the regeneration process was nearly completed by day 28 after injury (Figure 7B; D28). Individual fiber areas were measured at day 14 and day 28 to quantitatively assess muscle regeneration and fiber hypertrophy. The results indicated that the regenerated fibers in the muscles of lactate-treated mice were significantly larger than those in saline-treated mice (Figure 7D,E).

## 3. Discussion

The data obtained this study demonstrated that sustained treatment with lactate enhanced myogenic differentiation and gene expression of fast-twitch-type MHC. These effects would potentially lead to muscle fiber hypertrophy in vitro and in vivo. In addition, the results of our in vitro study suggested that the transcription factor MyoD is involved in the mechanism behind these lactate-induced effects. The C2C12 cell line used in our study was subcloned from a myoblast obtained from adult mouse muscle and can be induced to differentiate into contracting myotubes expressing muscle-specific proteins. Hence, it is generally considered to be a good model for muscle satellite cells. The effects of lactate on C2C12 myoblasts observed in this study might reflect the underlying mechanism by which exercise training, accompanied by increased serum lactate, induces muscle hypertrophy through satellite cell activation.

In in vitro experiments, we found that incubating C2C12 cells with lactate initially resulted in increased *Myod* gene expression, thereby inducing myogenic differentiation. Although we did not clarify the exact mechanism behind this, we speculate that lactate might affect a transcription network associated with MyoD, consistent with previous suggestions that lactate can regulate gene expression through a specific transcription network [33,34,35]. A previous report showed that *Myod* gene expression was greatly stimulated by MyoD itself, suggesting that its expression is controlled by a positive feedback loop [36]. In addition, Fulco et al. reported that the transcriptional activity of *Myod* was negatively regulated by Sir2, an NAD^+^-dependent deacetylase [37]. These findings led to our speculation that lactate causes a decreased [NAD^+^]/[NADH] ratio in muscle cells because of the lactate/pyruvate equilibrium. This would accelerate a MyoD autoloop through Sir2 inhibition. This hypothesis was supported by the time-dependent responses of *Myod* expression to lactate (Figure 2A), with lactate-induced increases in *Myod* expression being more evident at later stages of differentiation.

The expression of *Myog*, encoding myogenin, another member of the MRF family, was not affected by lactate, although its expression was also regulated by MyoD in another experimental setting [38]. Both MyoD and myogenin play important roles in controlling muscle differentiation and recognize similar sets of genes [38]. However, their distribution patterns in mature muscles are quite different. The MyoD transcript was mainly observed in fast-twitch muscles such as extensor digitorum longus (EDL) or TA muscles, whereas that of myogenin was found to be abundant in slow-twitch muscles such as the soleus muscle [39,40]. These observations suggest that regulatory systems in which individual MRFs are involved are muscle phenotype-specific, and that the effects of lactate involving MyoD observed in this study might occur predominantly in fast-type muscles.

In fact, lactate-induced *Myod* expression led to increased gene expression of MHC IIb and IIx, the predominant isoforms in fast-type muscle fibers [41,42], but not of MHC I and IIa. Recently, Nederveen et al. [43] observed increased *Myod* expression and higher numbers of MyoD-positive satellite cells after a single bout of exercise in young men who had undergone 16 weeks of resistance training. These effects were associated with a more marked increase in the cross-sectional area of type II fibers than in that of type I fibers. We speculate that elevated lactate levels during the training period might have been involved in this mechanism. Promoter assays showed that the specific E-box motif that is preferentially recognized by MyoD [30] was required for lactate-stimulated increases in MHC IIb gene promoter activity. This suggested that lactate enhanced MHC IIb gene expression at the transcriptional level through a network involving MyoD. Previous studies established that MyoD-dependent expression of MHC IIb was crucial for determining the fiber type in fast muscles and/or for a phenotypic shift in fiber type toward a faster character in slow muscles [30,44]. As atrophy occurs preferentially in the fast-twitch myofibers of aged muscles [45,46,47,48], the effects of lactate observed in our study might provide clues for an approach to prevent age-associated muscle atrophy.

Previous studies presented somewhat inconsistent results. For example, Willcomm et al. reported that repeated short-term (2 h) stimulation with 20 mM lactate for five days suppressed the expression of myogenin and MHC associated with inhibited p38 MAPK phosphorylation [22]. In contrast, Oishi et al. [24] showed that single treatment of C2C12 myotubes with 10 mM lactate for 6 h resulted in increased myogenin (but not MyoD) levels and activation of p70S6K, a critical regulator of muscle protein synthesis. Concomitant with these results, our data suggested that sustained, rather than short-term, exposure to elevated lactate affects MyoD expression during myogenic differentiation. As an in vitro model with lactate maintained at a constant level for a few days does not completely reflect the conditions in a physiological context, further work is required to assess the impacts of elevated lactate on MRF regulation. 

Accelerated muscle differentiation and increased muscle fiber size in response to lactate stimulation were observed in our animal study, similar to our in vitro findings. In our study, we used intraperitoneal lactate injection, not exercise loading, to evaluate the direct effects of lactate, while eliminating the potential influence of muscle contraction. Blood lactate levels were elevated after the injection of 500 mg/kg lactate, mimicking lactate kinetics after high-intensity (80–90% VO_2max_) exercise [25,49]. In histological examination of TA muscles, enhancement of the regeneration process following injury by the intramuscular administration of glycerol was clearly observed in mice injected with lactate, compared with that in mice not receiving lactate. In regenerating muscles exhibiting increased *Myod* expression, a previous study demonstrated downregulation of both monocarboxylate transporter (related to the efflux of lactate) and lactate dehydrogenase (LDH)-B (the enzyme for the conversion of lactate to pyruvate) [50]. This suggested an increased demand for lactate in growing muscle tissues expressing MyoD. Ours is the first study to evaluate the direct effects of lactate on muscle remodeling; the results suggested that lactate elevation, associated with high-intensity exercise, has favorable effects on muscle remodeling and hypertrophy, independent of the effects of the exercise itself. 

The present study had some limitations. First, the conditions involving a high lactate concentration for a long period in the in vitro studies were not physiologically relevant. We thus need to evaluate the effect of lactate using different concentrations and time periods in future examinations. Second, there were inconsistencies in the time points used for characterizing *Myod*. Although *Myod* expression peaked at day 3 in the C2C12 experiment, it was determined at day 7 in vivo. Its expression in vivo also peaked at around day 3 in the regeneration process [51,52], so MRF profiles should be determined at earlier stages. Third, the mouse TA muscles used in this study were composed only of IIb and IIx fibers [53]. The composition of fiber types was strongly dependent on the particular muscle type and animal species. Further experiments are thus needed to clarify whether lactate can induce muscle hypertrophy in other muscles and/or animals, including humans.

In conclusion, in this study, we demonstrated that a sustained elevation of lactate would support myogenic differentiation, as well as muscle fiber hypertrophy. Regarding the mechanism involved in this, our findings suggest a novel mechanism in which increased MyoD expression, facilitated by lactate, might play a critical role in the increase in predominantly fast-twitch-type MHCs. A notable finding is that the effect of lactate was probably enhanced through auto-regulatory loops associated with this molecule. Blood levels of lactate after exercise can reach those used in our study. Thus, our findings are potentially important for considering efficient exercise-based strategies to maintain muscle volume and/or to induce muscle hypertrophy.

## 4. Materials and Methods

### 4.1. Cell Culture and Differentiation

C2C12 myoblasts (RIKEN, Tsukuba, Japan) and primary myoblasts obtained from adult male mice (kindly provided by Dr. Yasuro Furuichi, Tokyo Metropolitan University) were cultured in Dulbecco’s modified Eagle’s medium (DMEM) containing 10% fetal bovine serum (FBS) at 37°C under a humidified atmosphere of 95% air and 5% CO_2_. The differentiation of myoblasts to myotubes was induced at a cell confluence level of 80–100% (day 0) by switching to a differentiation medium, DMEM with 2% horse serum. The medium was changed every day until cells were harvested for analysis. In some experiments, cells were continuously incubated in differentiation medium with sodium lactate (Otsuka Pharmaceutical, Tokyo, Japan) or sodium chloride (Otsuka Pharmaceutical) from day 0, unless otherwise indicated. The equivalent volume of distilled water was used as a vehicle control. The added lactate did not affect the pH of the medium. 

### 4.2. Gene Expression Analysis by Real-Time PCR

Total RNA was isolated using Sepasol-RNA I reagent (Nacalai Tesque, Kyoto, Japan) and first-strand cDNA was synthesized using a ReverTra Ace qPCR RT Master Mix (Toyobo, Osaka, Japan). Samples were run in 10 µL reactions using SYBR Premix Ex Taq II (Takara Bio, Shiga, Japan) on a Thermal Cycler Dice^®^ Real Time System (Takara Bio). Gene products were expressed in terms of mRNA levels and were normalized to a standard housekeeping gene (*Gapdh*) using the ΔΔ*C*T method. The primer sequences are shown in Supplemental Table S1.

### 4.3. Western Blotting

C2C12 cells were washed with phosphate-buffered saline (PBS) and dissolved in lysis buffer (1% Triton X-100, 0.45% sodium pyrophosphate, 100 mM NaF, 2 mM Na_3_VO_4_, 50 mM HEPES (pH 8.0), 147 mM NaCl, 1 mM EDTA, and protease inhibitor mixture (cOmplete; Sigma-Aldrich, Tokyo, Japan)) and centrifuged at 12,000× g for 15 min at 4 °C. The supernatant was used as the cell lysate. Cell lysate protein (20 μg per lane) was resolved on 7.5% [for myosin heavy chain (MHC)] or 10% (for other proteins) SDS-PAGE gel and the protein bands were then transferred to PVDF membranes (Hybond-P; GE Healthcare Life Science, Tokyo, Japan). The membranes were blocked with 5% BSA for 1 h at room temperature and incubated overnight at 4 °C with an antibody, as indicated, against each of the following: MHC (MF-20; Hybridoma Bank, Iowa, IA, USA), P70S6K (#9202S; Cell Signaling, Danvers, MA, USA), phosphorylated-P70S6K (#9205S; Cell Signaling), or β-actin (#sc47778; Santa Cruz Biotechnology, Dallas, TX, USA). MF-20 recognized all MHC isoforms. After the membranes were incubated with the appropriate secondary antibodies for 1 h at room temperature, the bands were visualized with an enhanced chemiluminescence system (Immunostar LD; Wako Chemical Industries, Tokyo, Japan) and scanned with an ImageQuant LAS-4000 (GE Healthcare Life Science) luminescence image analyzer. The optical density of each band was analyzed with ImageJ software (National Institutes of Health, Bethesda, MD, USA).

### 4.4. Immunofluorescence and Calculation of Fusion Index

Differentiated C2C12 cells were fixed with PBS containing 4% paraformaldehyde for 15 min at room temperature and permeabilized in 0.2% Triton X-100/PBS for 10 min. The fixed and permeabilized cells were then incubated with the MF-20 anti-MHC antibody overnight, washed with PBS, and incubated for 1 h with anti-mouse IgG conjugated with fluorescein isothiocyanate (FITC) (#115-095-062; Jackson ImmunoResearch, West Grove, PA, USA). After washing samples with PBS, the nuclei were stained with 4′,6-diamidino-2-phenylindole (DAPI). For each condition, 15 images of the cells (*n* = 3, 5 images per sample) were taken using a fluorescence microscope (IX71; Olympus, Tokyo, Japan). The cell number of each sample was counted from the photographs, and the MHC-positive cells containing three or more nuclei were defined as myotubes. The fusion index (%) was calculated using the following formula: (nuclei within myotubes/total number of nuclei) × 100.

### 4.5. Reporter Assays

Full-length mouse MyoD cDNA was amplified by PCR and cloned into a pcDNA3-based mammalian expression vector (pcDNA‒MyoD). A series of plasmids containing the luciferase gene under control of the *Myh4* (encoding myosin heavy chain IIb) gene promoter was constructed as follows. Promoter sequences of *Myh4* (−1347 to +33) were amplified from mouse genomic DNA using specific primers with suitable restriction sites. They were cloned into the pGL3 basic vector (Promega, Tokyo, Japan). Deletion constructs were prepared from this construct using endogenous restriction sites or a PrimeSTAR Mutagenesis Basal Kit (Takara Bio). C2C12 cells (6 × 10^3^ to remain undifferentiated or 6 × 10^4^ for differentiation) were seeded into 24-well plates. Then, for differentiated samples only, the growth medium was changed to differentiation medium the next day. The indicated plasmid was then transiently transfected into cells using Lipofectamine 3000 Transfection Reagent (Thermo Fisher Scientific, Yokohama, Japan), in accordance with the manufacturer’s protocol. *Renilla* luciferase control vectors were co-transfected to control for transfection efficiency. Cells were lysed and luciferase activity was measured with the Dual-Luciferase Reporter Assay System (Promega, Tokyo, Japan) and expressed as fold induction, corrected for transfection efficiency using *Renilla* luciferase activity.

### 4.6. Animal Care

ICR male mice (eight weeks of age, *n* = 18; Japan SLC, Hamamatsu, Japan) were used. Mice were randomly assigned to two groups: a control group (Con; *n* = 9) and a lactate group (Lac; *n* = 9). They were injected with 50 µL of 50% *v*/*v* glycerol into the bilateral tibialis anterior (TA) muscles, as previously described [54,55], under anesthesia by isoflurane inhalation. Then, sodium lactate (500 mg/kg) or the equivalent volume of saline was administered daily by intraperitoneal injection for seven days to the mice in the Lac and Con groups, respectively. At day 7, 14, and 28 after glycerol injection, mice were sacrificed (three mice for each time point) and TA muscles were harvested. Muscle tissues were immediately frozen and cryosectioned (7 µm thickness) at the mid-belly region, followed by staining with hematoxylin and eosin. Twelve representative fields per animal (four fields from three sections) were examined and the myofiber areas in the samples, determined at day 14 and 28 after glycerol injection, were analyzed using a digital microscope (BZ-X700; Keyence, Osaka, Japan). All animal procedures were approved by the Animal Ethics Committee of Ochanomizu University (Approval No.: 17021R, Approval date: 4/8/2017) and performed in accordance with the Japanese Act on Welfare and Management of Animals. 

### 4.7. Statistical Analysis

All cell experiments were performed at least twice independently. The results are expressed as means ± standard error (SE). For statistical analyses of data from cellular experiments, an unpaired *t* test, a one-way analysis of variance, or a two-way analysis of variance was used, as appropriate. A nonparametric test (Wilcoxon) was used to compare fiber areas in mouse muscle samples. All analyses were performed using IBM SPSS Statistics software for Windows, Version 24.0. (IBM Corp., Armonk, NY, USA). Differences of *p* < 0.05 were considered statistically significant.

## Supplementary Materials

Supplementary materials can be found at http://www.mdpi.com/1422-0067/19/11/3649/s1.

## Abbreviations

EDL	extensor digitorum longus (muscle)
FBS	fetal bovine serum
MHC	myosin heavy chain (protein)
MRF	myogenic regulatory factor
Myf5	myogenic factor 5
Myh	myosin heavy chain (gene)
MyoD	myogenic determination protein
SOL	soleus (muscle)
TA	tibialis anterior (muscle)
