# Supplementary material for: Lactate Promotes Myoblast Differentiation and Myotube Hypertrophy via a Pathway Involving MyoD In Vitro and Enhances Muscle Regeneration In Vivo
Source: Int J Mol Sci. 2018 Nov 19;19(11):3649. doi: 10.3390/ijms19113649 (PMC6274869; doi:10.3390/ijms19113649)
Supplement: Supplementary file 1 [file ijms-19-03649-s001.pdf]

Table S1. Sequences of primers used for real-time PCR

Gene and encoded protein names		
Myod	Forward	CCCCGGCGGCAGAATGGCTACG
(MyoD)	Reverse	GGTCTGGGTTCCCTGTTCTGTGT
Myf5	Forward	GAGGGAACAGGTGGAGAACTATTA
(Myf5)	Reverse	CGCTGGTCGCTGGAGAG
Myog	Forward	ACTCCCTTACGTCCATCGTG
(Myogenin)	Reverse	CAGGACAGCCCCACTTAAAA
Myh4	Forward	CCGAGCAAGAGCTACTGGA
(MHC IIb)	Reverse	TGTTGATGAGGCTGGTGTTC
Myh7	Forward	CCAAGGGCCTGAATGAGGAG
(MHC I)	Reverse	GCAAAGGCTCCAGGTCTGAG
Myh2	Forward	AAGCGAAGAGTAAGGCTGTC
(MHC IIa)	Reverse	TGATTGCTTGCAAAGGAAC
Myh1	Forward	AGGCCAGGGTCCGTGAA
(MHC IIx)	Reverse	CCACGTTGCGCTTCTGTTC
Gapdh	Forward	TGACGTGCCGCCTGGAGAAA
(GAPDH)	Reverse	AGTGTAGCCCAAGATGCCCTTCAG

MHC; myosin heavy chain, GAPDH; glyceraldehyde-3-phosphate dehydrogenase
